# RT-qPCR half-reaction optimization for the detection of SARS-CoV-2

**DOI:** 10.1590/0037-8682-0319-2020

**Published:** 2021-12-17

**Authors:** Priscila Lamb Wink, Fabiana Volpato, Daiana de Lima-Morales, Rodrigo Minuto Paiva, Julia Biz Willig, Hugo Bock, Fernanda de Paris, Afonso Luís Barth

**Affiliations:** 1 Hospital de Clínicas de Porto Alegre, Centro de Pesquisa Experimental, Laboratório de Pesquisa em Resistência Bacteriana, Porto Alegre, RS, Brasil.; 2 Hospital de Clínicas de Porto Alegre, Laboratório de Diagnóstico de SARS-CoV-2, Porto Alegre, RS, Brasil.; 3 Universidade Federal do Rio Grande do Sul, Faculdade de Farmácia, Programa de Pós-Graduação em Ciências Farmacêuticas, Porto Alegre, RS, Brasil.; 4 Universidade Federal do Rio Grande do Sul, Faculdade de Medicina, Programa de Pós-Graduação em Ciências Médicas, Porto Alegre, RS, Brasil.; 5 Hospital de Clínicas de Porto Alegre, Programa de Residência Multiprofissional, Porto Alegre, RS, Brasil.

**Keywords:** Coronavirus, Coronavirus disease 2019, COVID-19, RT-qPCR half-reaction, SARS-CoV-2

## Abstract

**INTRODUCTION:**

The main laboratory test for the diagnosis of coronavirus disease 2019 (COVID-19) is the reverse transcription real-time polymerase chain reaction (RT-qPCR). However, RT-qPCR is expensive because of the number of tests required. This study aimed to evaluate an alternative to the RT-qPCR approach for the detection of sudden acute respiratory syndrome coronavirus 2 (SARS-CoV-2) that is half of the total volume currently recommended by the US Centers for Disease Control and Prevention.

**METHODS:**

The analytical limit of detection (LoD) and the reaction efficiency using half volumes of the RT-qPCR assay were evaluated for the N1 and N2 regions using a synthetic control RNA. A panel of 76 SARS-CoV-2-positive and 26 SARS-CoV-2-negative clinical samples was evaluated to establish clinical sensitivity and specificity.

**RESULTS:**

The RT-qPCR assay efficiency was 105% for the half and standard reactions considering the N2 target and 84% (standard) and 101% (half) for N1. The RT-qPCR half-reaction LoD for N1 and N2 were 20 and 80 copies/µL, respectively. The clinical sensitivity and specificity were 100%. The half reaction presented a decrease of up to 5.5 cycle thresholds compared with standard RT-qPCR.

**CONCLUSIONS:**

The use of the RT-qPCR half-reaction proved feasible and economic for the detection of SARS-CoV-2 RNA.

## INTRODUCTION

Coronavirus disease 2019 (COVID-19), caused by the novel severe acute respiratory syndrome coronavirus 2 (SARS-CoV-2) virus, emerged in Wuhan, China, at the end of 2019 and has rapidly spread worldwide. This pandemic has resulted in more than 224 million cases worldwide and around 4.6 million confirmed deaths requiring unprecedented public health action^1^ ^,^ ^2^. No proven effective therapy is currently available for SARS-CoV-2, which reinforces the importance of massive testing and quarantining exposed persons to limit its spread^3^. 

Reverse transcriptase polymerase chain reaction (RT-qPCR) is the main method used to detect SARS-CoV-2^2^. Distinct RT-qPCR testing protocols comprising different probes and primers used in a multi-step PCR workflow were swiftly established and made publicly available by the World Health Organization and the Centers for Disease Control (CDC)^4^ ^,^ ^5^. 

Considering the high infectivity of SARS-CoV-2, it is very important to increase the RT-qPCR testing rate to allow the fast and accurate identification of infected people to prevent viral dissemination. However, reagents are expensive; thus, there is a need for new protocols to reduce the costs of RT-qPCR. 

 This study aimed to optimize the RT-qPCR method for the detection of SARS-CoV-2 using only half of the total volume of reagents currently recommended by the CDC protocol. We also tested a panel of 76 SARS-CoV-2-positive and 26 SARS-CoV-2-negative clinical samples using the standard assay to preliminarily evaluate the clinical sensitivity and specificity of the half reaction. 

## METHODS

### Nucleic acid extraction 

RNA was extracted from 600-μL respiratory specimens using the Abbott mSample Preparation System (Promega, Madison, WI, USA) with an Abbott M2000 instrument (Abbott, Chicago, IL, USA) following the manufacturer’s instructions. Total nucleic acids were eluted in 80 μL of Abbott mElution buffer. The study was approved by the Ethics Committee of Hospital de Clínicas de Porto Alegre (CAAE: 30767420.2.0000.5327).

### Reverse transcriptase quantitative polymerase chain reaction 

Two genes of the nucleocapsid protein (N), N1 and N2, were amplified using a set of primers and probes as described by the CDC (USA) RT-qPCR assay. Primers and probes were purchased from Integrated DNA Technologies (Coralville, IA, USA). We performed both the standard RT-qPCR and RT-qPCR half reaction assays in parallel using the SuperScript™ III One-Step RT-PCR System (Thermo Fisher Scientific, Waltham, MA, USA). A description of the RT-qPCR assay is available on the CDC Laboratory Information website for COVID-19^6^. Briefly, to perform the standard RT-qPCR reaction, a 5-μL isolated RNA sample was mixed with 15 μL of one-step RT-qPCR mix containing 10 μL of 2× Master Mix, 0.4 μL of platinum enzyme, 1.5 μL each of 10 μM combined primer/probe mix, 0.4 μL of 2.5 μM ROX passive reference dye, and 2.7 μL of water. For the RT-qPCR half reaction, half of the volumes of the reagents listed above were used without dilution. Therefore, our proposed reaction included 6 μL of reagents and 4 μL of RNA, totaling 10 μL in each well, which resulted in a reaction with more template RNA in relation to the reaction mixture (4-6 μL compared to 5-15 μL from the standard RT-qPCR reaction). 

We used three control samples in each RT-qPCR run: a positive template control, water as a negative control, and an internal control (human ribonuclease P gene, RNAse P). We conducted both assays (standard and half reactions) in 96-well plates using an Applied Biosystems QuantStudio Real-Time PCR 3 Instrument (Thermo Fisher Scientific). Cycling conditions were as follows: 30 min at 50°C for reverse transcription, 2 min at 95°C for activation of the platinum enzyme, and 40 cycles of 15 s at 95°C and 35 s at 55°C. We used a threshold that was automatically established by the equipment. 

A cycle threshold (Ct) value lower than 40 for the N1 and N2 targets was reported as RT-qPCR positive. The result was considered negative if the Ct was undetectable or greater than 40. RNAse P was used to monitor nucleic acid extraction, specimen quality, and the presence of reaction inhibitors^6^.

### Standard curve to assess efficiency 

Assay specificity was determined using high-titer virus stock as well as SARS-CoV-2-positive clinical samples using the standard RT-qPCR assay in a Quant Studio Real-Time PCR system (Thermo Fischer Scientific). The reaction efficiency was validated for the N1 and N2 targets using a standard curve with five points with a serial dilution (from 1 × 10^5^ to 10 copies/µL) of a synthetic control RNA (Integrated DNA Technologies) in triplicate. The Ct values of the serial dilutions were plotted against the target concentration (number of viral copies). We determined the slope of the curve by linear regression and defined the required levels for PCR efficiency ([100 × 10^-^ ^1^ ^/slope^ - 1]) and linearity (*R* ^2^) of each RT-qPCR target to be 90-110% and >0.95, respectively^7^. Data were automatically calculated using the Thermo Fisher cloud dashboard.

### Limit of detection of RT-qPCR with N1 and N2 

To determine the analytical limit of detection (LoD) of the RT-qPCR half reaction assay, we tested 1, 5, 10, 20, 50, 80, and 100 copies of SARS-CoV-2 RNA per microliter. The LoD of the N1 and N2 targets was independently assessed using serially diluted synthetic control RNA. The calibration curve for the genomic copy number versus Ct value was obtained using the Quant Studio RT-qPCR instrument (Thermo Fischer Scientific). A series of 20 parallel reactions per concentration step was prepared and tested by RT-qPCR. 

Assay reproducibility was tested using replicated dilutions of the RNA transcripts, and intra- and inter-assay variabilities were evaluated for each dilution point on different days.

### Evaluation of clinical specimens 

A total of 102 clinical samples from different patients attending the Hospital de Clínicas de Porto Alegre in Southern Brazil were obtained by oronasopharyngeal swabbing. All samples were analyzed using the standard and half reaction protocols described above. 

## RESULTS

### Standard curve to assess reaction efficiency 

**FIGURE 1:**
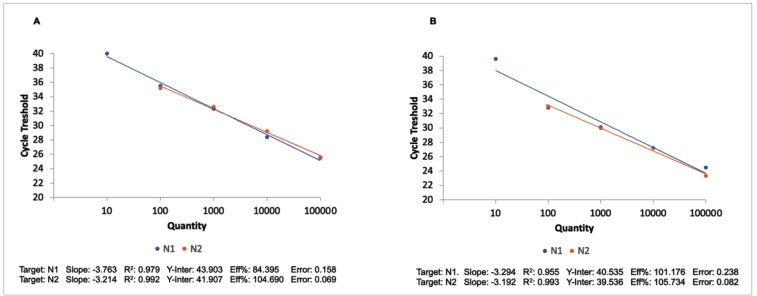
Determination of detection efficiency for the N1 and N2 assays. A) RT-qPCR standard curve plot; B) RT-qPCR half reaction standard curve plot.

The reaction efficiency using half of the assay volume was evaluated for the N1 and N2 regions versus the standard RT-qPCR reaction using a synthetic control RNA. The RT-qPCR efficiencies for the half reaction were 101.2% and 105.7% for the N1 and N2 targets, respectively. Conversely, for the standard RT-qPCR reaction, the efficiency values were 84.4% (N1) and 104.7% (N2) (Table 1). The *R* ^2^ for each target was higher than 0.95 for both reactions (Figure 1).

**TABLE 1:** Efficiency of the two RT-qPCR approaches using a dilution series of a synthetic control RNA.

		Mean Ct value^c^						
Target		10^5^ copies/µL	10^4^ copies/µL	10^3^ copies/µL	10^2^ copies/µL	10^1^ copies/µL	Slope^d^	Efficiency (%)^e^
N1	SdRT-qPCR a	25.5	28.4	32.2	35.5	41.4	-3.763	84.4
	hRT-qPCR b	24.5	27.2	30.1	32.8	39.6	-3.294	101.2
N2	SdRT-qPCR	25.6	29.2	32.6	35.2	ND f	-3.214	104.7
	hRT-qPCR	23.3	27.2	30.0	33.0	ND	-3.192	105.7

a SdRT-qPCR = Standard RT-qPCR

b hRT-qPCR = RT-qPCR half reaction

c Values shown are the means of triplicate tests.

d Slope = Y intercept - slope log10

e Efficiency = [100 × 10^(-1/slope)^ -1]

f ND: not detected

Eff%: curve efficiency; N1: N1 target; N2: N2 target; R^2^: curve linearity.

Although we planned to generate efficiency curves for the RT-qPCR half and standard reactions using five dilution points for both targets (N1 and N2), this was only possible for the N1 target. Because of the LoD of the N2 target, we were able to obtain a Ct value for only four dilution points (from 1 × 10^5^ to 1 × 10^2^ copies/µL). 

### LoD of RT-qPCR for N1 and N2 

Serial dilutions of the nucleocapsid RNA transcripts were tested to assess the detection limits and dynamic ranges of the RT-qPCR assays. The lower LoD was 20 transcript copies per reaction for the N1 target versus 80 copies per reaction for the N2 target. 

At these lower copy detection limits for the N1 and N2 targets, the assay reproducibility was 95% and 100%, respectively. Eighty-five percent reproducibility was achieved at the dilution that contained 10 transcript copies per reaction with the N1 target and at the 50 transcript copies per reaction dilution with the N2 target.

### Evaluation of clinical specimens 

The RT-qPCR half reaction assay was performed of 102 clinical samples. All 102 samples were subjected to the standard RT-qPCR described by the CDC; of them, 76 tested positive, with Ct values varying from 14.9% to 33.7%. The half reaction assay yielded positive results for all 76 specimens. Likewise, none of the 26 specimens with negative results on the standard reaction presented positive results on the half reaction assay.

The Ct values of the half and standard reactions of RT-qPCR were compared to quantitatively analyze the results. The half-reaction assay showed a decrease in Ct values for 55 and 73 clinical specimens for the N1 and N2 targets, respectively. The average decrease in Ct values was 0.7 and 2.8 for the N1 and N2 targets, respectively, versus the standard RT-qPCR (Table 2). 

**TABLE 2:** Comparison of Ct^a^ values between the standard and half reactions of RT-qPCR for SARS-CoV-2-positive clinical samples that presented a decrease in Ct.

	Average of SdRT-qPCR b minus hRT-qPCR c	
RT-qPCR Ct values	N1	N2
≤20	0.5 ± 0.4 (*n* = 20)	2.3 ± 0.8 (*n* = 16)
20-25	0.8 ± 0.5 (*n* = 12)	2.6 ± 0.8 (*n* = 21)
25-30	0.7 ± 0.5 (*n* = 16)	2.7 ± 1.0 (*n* = 16)
>30	0.7 ± 0.3 (*n* = 7)	3.5 ± 1.3 (*n* = 20)
**All clinical samples**	**0.7 ± 0.4 (*n* = 55)**	**2.8 ± 1.1 (*n* = 73)**

a Ct = cycle threshold

b SdRT-qPCR = standard reaction RT-qPCR

c hRT-qPCR = half reaction RT-qPCR

Twelve isolates with an increased Ct value for the N1 target (average, 0.4) and two isolates for the N2 target (0.1) were observed for the half RT-qPCR assay versus the standard reaction. Nine isolates presented no difference in the Ct values for the N1 target and only one for the N2 target.

## DISCUSSION

Here we evaluated RT-qPCR using half of the total reaction volume currently recommended by the US CDC protocol to detect SARS-CoV-2. If the half reaction assay proved to be a reliable method of detecting the RNA of SARS-CoV-2 would save consumables. We found that the RT-qPCR half reaction efficiency was equivalent to that of the standard RT-qPCR reaction for the N2 target. Moreover, we observed an important increase in efficiency (from 84.4% to 101.2%) for the N1 target using the RT-qPCR half reaction. The LoD for N1 and N2 was also evaluated for the RT-qPCR half reaction assay, and we found that N1 was more sensitive than N2 (LoD = 20 and 80 copies/µL, respectively). 

When we compared the Ct values of the SARS-CoV-2-positive clinical samples obtained by the two assays, we observed a decrease in the values of Ct for most samples, either for the N1 (55 cases [72%]) or N2 (73 cases [96%]) targets in the half RT-qPCR assay. These findings indicate that the half-reaction assay yields a better yield than the standard reaction. 

The data mentioned above indicate that a reduction in the reaction volume does not influence the quality of the results; rather, we found an increase in the sensitivity and efficiency of the technique. As the RNA volume was kept the same as the original protocol while all other reagents were reduced, it is possible that the increased ratio of RNA in the final reaction mixture would be responsible, at least partially, for the increased sensitivity and efficiency of the RT-qPCR half reaction.

Furthermore, the comparison of Ct values of the two reaction approaches with clinical samples showed that the kinetics of sensitivity depended on the viral load using the Ct value. The lower the Ct value, the smaller the differences between the approaches (Table 2).

Although RT-qPCR assays remain the molecular test of choice for the diagnosis of SARS-CoV-2 infection, considerable efforts have been made to improve the detection rate, and a variety of improved or new approaches have been developed. Since RT-qPCR methods require time-consuming sample handling and post-PCR analysis, immunoassays have been developed for the rapid detection of SARS-CoV-2 antigens or antibodies (immunoglobulin M or G) to COVID-19. They would theoretically provide the advantage of a fast time to results and the low-cost detection of SARS-CoV-2 but are likely to suffer from poor sensitivity in the early infection period^8^.

To enable massive coronavirus testing, the pooling of clinical samples was proposed as a testing strategy that would significantly increase the testing capacity of laboratories^9^ ^,^ ^10^. However, the use of pooling would be much more effective in testing clinical samples in scenarios with a low prevalence of SARS-CoV-2, as a positive result for the pool would require all samples to be tested individually^9^ ^,^ ^10^. Furthermore, as previously described, considering that the concentrations of RNA were reduced in the pooled specimens, the Ct values were expected to increase by five Ct values versus single samples^9^. Accordingly, pools with a high number of samples (30 samples) may present false-negative results on RT-qPCR for SARS-CoV-2, especially when a positive sample has a higher Ct^9^.

The present study optimized the RT-qPCR method for the detection of SARS-CoV-2, with the half reaction presenting better performance as well as higher sensitivity and specificity compared to the standard reaction. It would be hugely advantageous to adapt the RT-qPCR protocol in molecular diagnostic laboratories, as it is essential for economic purposes to save reagents and increase the number of patients tested for COVID-19 worldwide. Moreover, decreasing the total reagent volume and increasing the template concentration for a single run would be very useful for increasing test sensitivity. In light of the current situation, the availability of a one-step PCR protocol that achieves both high sensitivity and specificity would be beneficial for facing the SARS-CoV-2 pandemic.
